# One Health—Key to Adequate Intervention Measures against Zoonotic Risks

**DOI:** 10.3390/pathogens12030415

**Published:** 2023-03-06

**Authors:** Dana A. Thal, Thomas C. Mettenleiter

**Affiliations:** Friedrich-Loeffler-Institut, Federal Research Institute for Animal Health, 17493 Greifswald-Insel Riems, Germany

**Keywords:** zoonoses, One Health, OHHLEP, AMR, influenza A virus, West Nile virus

## Abstract

Zoonotic diseases are a heterogenous group of infections transmittable between humans and vertebrate animal species. Globally, endemic and emerging zoonoses are responsible for high social and economic costs. Due to the particular positioning of zoonoses at the human-animal-environment interface, zoonotic disease control is an integral part of One Health, which recognizes the close link between human, animal and ecosystem health. During recent years, the validity of the One Health approach has been recognized by academia and policy makers. However, gaps are still evident, particularly in the implementation of the concept as a unifying, integrated approach for different sectors and disciplines for the control of zoonoses. For example, while cooperation between human and veterinary medicine has made significant progress, networking with environmental sciences leaves room for improvement. Examination of individual intervention measures can help to gain valuable insights for future projects, and help to identify existing gaps. This is also a task for the One Health High-Level Expert Panel, which was established by WHO, OIE, FAO and UNEP to give science-based strategic advice on One Health measures. Overall, we should aim to learn from current situations, and to identify the best practice examples available, to continuously develop and improve One Health concepts for the control of zoonoses.

## 1. Introduction

Many infectious diseases can be transmitted between humans and non-human animal species, referred to as zoonoses. Zoonoses are a heterogeneous group of infections caused by a wide range of pathogens (bacteria, viruses, fungi, parasites, prions) using different transmission routes (e.g., vector-borne, direct transmission, food-borne), and leading to a variety of symptoms and disease severities. A look back into human history, using paleopathological studies and ancient DNA analyses, suggests that zoonoses such as brucellosis may have occurred as early as 2.4 to 2.8 million years ago [1]. Today the growing human population is confronted with an increasing threat from endemic and re-/emerging zoonoses, that cause a significant burden of disease at local, regional and global levels, and are associated with substantial social and economic costs [2,3].

Zoonotic infections are an integral part of human history. However, it has been stated that the rate of emergence of zoonoses has increased in recent decades [4], which has been linked to anthropogenic influences such as population size [5], climate change [6], mobility, global trade, intensive livestock farming [7], land use changes [8] and biodiversity loss [9], among others. This increased occurrence poses a risk to human and animal health alike. Because zoonotic disease transmission cycles link ecosystems, humans, and animals, they are a classic example for the need of One Health approaches. The One Health approach recognizes the close connection between ecosystems, humans and animals and seeks holistic intervention concepts to optimize the well-being of all.

The One Health concept is not new, and many different definitions of the term One Health have been suggested over the years [10,11]. The SARS-CoV-2 pandemic brought renewed attention to the concept, and increased interest in translating it into action. Within this context, a comprehensive definition of One Health was recently published by the One Health High-Level Expert Panel (OHHLEP) [12]. It goes beyond recognizing the interconnectedness of health systems, by suggesting how collaboration, coordination, communication and capacity building within health sectors, disciplines and society should be designed to achieve sustainably balanced health outcomes for all sectors. Key principles underlying the OHHLEP’s One Health definition are equity between all actors, social and cultural parity, human stewardship, recognition of the value of all living beings and the integration of different forms of knowledge and perspectives in transdisciplinary and multisectoral collaborations [12]. The new concept is characterized by moving away from an anthropocentric focus, towards a more holistic view of health. In line with the One Health definition, the Quadripartite Organizations—the Food and Agriculture Organization of the United Nations (FAO), the United Nations Environment Program (UNEP), the World Organization for Animal Health (WOAH), and the World Health Organization (WHO)—published a One Health Joint Plan of Action (OH JPA) that provides guidelines for the implementation of One Health actions by the four organizations together [13]. Furthermore, the OH JPA is intended to offer advice to national action plans, as well as to facilitate the optimization of existing initiatives.

## 2. Materials and Methods

Although the OHHLEP’s One Health concept goes beyond the control of zoonotic diseases and antimicrobial resistance, these represent important One Health topics which are also mirrored in the OH JPA’s action tracks. To support this assumption, the opportunities, and the need for One Health—taking the new OHHLEP definition as guidance—are presented in the following three different zoonotic pathogens, used as examples: airborne influenza A virus, vector-borne West Nile virus and the increasing number of antimicrobial-resistant zoonotic bacteria.

## 3. Results

### 3.1. Influenza A Virus

Influenza A viruses (IAVs) are a diverse group of viruses that occur in birds, as well as in humans and other mammalian species. Their broad host range and their genomic flexibility, as well as their airborne transmission capability, contribute to their pandemic potential [14]. In particular, the (re-) emergence of antigenic variants from animal reservoirs, which has also been discussed as the origin of the 1918 IAV pandemic [15], is a major concern [16]. IAVs naturally circulate in aquatic bird species. From there, the virus can be transmitted to other wildlife or livestock such as poultry or swine, where they adapt by mutation. With the intensification of global livestock production, the interfaces for interspecies transmissions, as well as the reservoir population for IAVs, have increased. This development might facilitate the rise of new pandemic virus strains [17]. For example, a European study demonstrated the presence of different swine influenza A viruses (swIAVs) in swine holdings, which can give rise to new, possibly zoonotic, IAV strains by reassortment with human IAVs [18]. The situation emphasizes the need for One Health interventions on multiple levels. Firstly, there is a need for monitoring and surveillance systems for IAVs, which should target wildlife, livestock and humans. Coordination and data sharing between these sectors might facilitate quick responses. Secondly, it concerns compliance with hygiene standards and general biosafety measures (e.g., wearing personal protective equipment), as well as appropriate management of livestock. This is not only advised from an economic point of view, but also with regard to the health protection of wildlife populations and humans. Thirdly, it concerns vaccination strategies to reduce the circulating virus load in human and animal populations, to minimize the risk of newly emerging strains and to protect vulnerable groups. IAVs can be transmitted over long distances by migratory birds, livestock trade or travel, making them a good example of a global problem that affects many stakeholders, cultures and ecosystems. This makes a One Health approach all the more reasonable.

### 3.2. West Nile Virus

Another example of the need for One Health concepts in zoonoses control are arthropod-borne diseases, such as the mosquito-borne Flavivirus West Nile virus (WNV). This virus is maintained in a sylvatic cycle between bird species and mosquitos. However, it can be transmitted to mammalian species, such as humans and horses, who are dead-end hosts. The spread of WNV to new geographic regions has been linked to globalization, land-use changes [8] and global warming, which provide the virus with favorable environmental conditions in new ecosystems [19]. This demonstrates how anthropogenic environmental changes can affect pathogen abundance, and highlights the important link between zoonotic pathogens and their environment. WNV can be lethal to both humans [20] and animals [19]. Therefore, collaborative surveillance systems and information sharing between disciplines can be beneficial to human medicine, as well as the veterinary sector, for the prevention and the control of disease outbreaks [19]. Italy has set up a WNV Surveillance Plan, that includes an integrated human and veterinary surveillance system that targets humans, horses, birds, and mosquitoes [21]. Even though infections in animals (birds, mosquitoes, horses and poultry) and human cases are entered into two different databases, a real-time data exchange protocol exists between the two systems [21]. This information sharing allows the sectors to take timely preventive measures, such as increased monitoring activities, vector control measures, vaccination campaigns for horses, or increased precautions for blood donations and organ transplantations [22]. It was estimated that the One Health approach to monitor WNV in Italy had significant cost benefits in comparison to a uni-sectoral approach, underlining the economic incentives to advance One Health approaches [23].

Taking the OHHLEP’s One Health definition as a guideline, WNV intervention strategies should include all stakeholders, including the public. Here, information campaigns targeting vulnerable groups might be beneficial to prevent cases or detect them at an early stage. Moreover, a stronger involvement of environmental sciences would allow for the inclusion of geographical and meteorological data to predict potential hotspots or new endemic areas due to alterations in climatic conditions [24]. Additionally, control measures should be evaluated not only for their direct effects on disease abundance, but also for their effects on other parameters in an ecosystem. Vector control measures, for example, should not only be evaluated for their efficiency to reduce the abundance of the vector, and consequently the pathogen, but also for their impact on other species in ecosystems.

### 3.3. Zoonotic Bacteria and AMR

Apart from zoonotic viruses, zoonotic bacteria are a major health concern globally. In the context of bacterial zoonoses and One Health, the fight against antimicrobial resistance (AMR) is a prime focus. The rise of pathogens with increased antimicrobial resistance has been declared by the World Health Organization as one of the top 10 global public health threats facing humanity [25]. Antimicrobials are substances that kill or inhibit the growth of microbes, such as the antibiotic penicillin against the bacterium *Escherichia coli* or the antifungal Fluconazole against the fungus *Aspergillus* [26]. Consequently, they are used in human medicine, as well as in veterinary medicine and agriculture. Although the existence of AMR predates the clinical use of antibiotics [27], their widespread application drives the increase in antimicrobial resistance [28,29]. Via wastewaters, AMR can also spread into, and within the environment [30]. Of particular concern are high resistance rates to antimicrobials, which are important for the treatment of humans and animals. In 2018 the WHO published a list of antibiotic-resistant bacteria to be prioritized in drug research [31]. Critical-priority bacteria from that list are carbapenem-resistant *Acinetobacter baumannii* and *Pseudomonas aeruginosa*, and carbapenem-resistant and third-generation cephalosporin-resistant *Enterobacteriaceae*. *Escherichia coli*, which belong to *Enterobacteriaceae,* are zoonotic and can additionally be found in various environmental samples [32]. The emergence and the spread of AMR genes among humans, animals, and the environment (including plants, water, soil and air) requires a holistic approach, to minimize negative impacts in all settings, and to develop appropriate monitoring and intervention approaches.

To reduce the risks of AMR, different intervention measures need to be considered: (a) optimization of antibiotics use, i.e., antibiotic stewardship; (b) appropriate waste management and hygiene standards; (c) development of new therapeutics and vaccines; (d) adequate supply and (regulated) access to antibiotics and diagnostic capacities; (e) global monitoring systems with high-quality data availability; (f) improved communication strategies. Most of these measures are being addressed in principle in many countries. However, the unequal distribution of resources between countries and sectors prevents a coordinated global approach. Especially low- and middle-income countries (LMICs) often lack adequate health care and sanitation systems to ensure the responsible use of antibiotics. In addition, education regarding the responsible use of antibiotics by health workers, farmers, patients and veterinarians, and clear regulation on the use of antibiotics are insufficient in some countries and need to be improved [33]. The problem of AMR is increased by the fact that the incentive to develop new antibiotics is low for the pharmaceutical industry, as it is not excessively profitable.

These necessary interventions cannot be addressed by one country or sector on its own. Therefore, the fight against AMR requires a classic One Health approach that calls for strong leadership, political cooperation and transdisciplinary approaches. 

## 4. Discussion

The three examples demonstrate the complex interdependencies of zoonotic infectious diseases at the human-animal-environmental interface and the legitimacy of the One Health concept in this area. Therefore, the One Health concept should form the basis for the design of intervention measures against zoonotic diseases. However, the examples also demonstrate that the biological and socio-economic background of each pathogen are unique. Consequently, there is no single blueprint for a One Health solution that fits every zoonotic pathogen. Rather, the OHHLEP’s One Health definition provides a novel perspective and places One Health in the larger context. In combination with the OH JPA, it provides a toolbox from which suitable intervention methods can be taken and modified to fit the specific situation. In practice, this could include sharing data between disciplines to implement robust surveillance and modelling systems [34], or close collaboration between human and veterinary medicine in vaccination campaigns [35]. A collaborative One Health approach begins with fundamental research projects that investigate the interactions between pathogen, vector, host and environment. This knowledge is required to understand underlying mechanisms, and to identify potential threats. Looking at the current One Health research landscape, it will be necessary to strengthen collaboration, particularly with environmental research [36], to better understand the impact of parameters such as biodiversity, climate change, land use changes or pesticide use on zoonotic infections. In addition, the impact of human and animal pathogens on ecosystems needs to be assessed. At this point, collaboration with biologists and wildlife experts is essential. A close exchange with these disciplines can also help to identify risks for, and prevent spill-over events. Furthermore, collaboration with various stakeholders from politics and society is needed to raise awareness of zoonotic pathogens, provide adequate education to affected communities, build appropriate diagnostic capacities, strengthen global cooperation and implement resilient health care systems. Last but not least, intervention measures must be adapted to local needs [37], and socio-economic, as well as cultural aspects have to be considered. In this context, social sciences are important in One Health interventions, especially when it comes to risk communication strategies and education programs, for instance, to prevent spillover events.

## 5. Conclusions

The health of humans strongly depends on a healthy environment, which includes animals and plants, as well as water, air and soil. Moving away from an anthropocentric view of zoonotic diseases opens new pathways to address these issues in more holistic and sustainable ways. Given the historic and current importance of zoonotic diseases, and the shared biology between humans and non-human animals, it is unlikely that we could completely eliminate the threat of zoonotic infectious agents. Thus, we should acknowledge this interconnectivity and base our intervention measures on the insight in line with the One Health concept. Although holistic intervention measures can be costly and time-consuming, joining forces also opens the path to cost-effective measures. Especially in low- and middle-income countries (LMICs) a profitable benefit-cost ratio might be a strong argument for One Health interventions [38]. Most strikingly, the SARS-CoV-2 pandemic has shown that the cost of just one (emerging) zoonotic infection can easily outweigh investments in prevention and control.

## Data Availability

Not applicable.
